# Heterocyst-infecting chytrid parasites reduce nitrogen fixation and host growth under nitrogen-limiting conditions in the cyanobacterium *Dolichospermum sp*

**DOI:** 10.1093/plankt/fbaf054

**Published:** 2025-10-08

**Authors:** Xujian Xu, Joren Wierenga, Mridul K Thomas, Bastiaan W Ibelings

**Affiliations:** Dazhou Key Laboratory of Agricultural Resources Development and Ecological Conservation in Daba Mountain, Sichuan University of Arts and Science, Tashi Road 519, Dazhou 635000, Sichuan, China; Department F.-A. Forel for Environmental and Aquatic Sciences and Institute for Environmental Sciences, University of Geneva, 66 boulevard Carl-Vogt, Geneva 1211, Geneva, Switzerland; Department F.-A. Forel for Environmental and Aquatic Sciences and Institute for Environmental Sciences, University of Geneva, 66 boulevard Carl-Vogt, Geneva 1211, Geneva, Switzerland; Department F.-A. Forel for Environmental and Aquatic Sciences and Institute for Environmental Sciences, University of Geneva, 66 boulevard Carl-Vogt, Geneva 1211, Geneva, Switzerland; Department F.-A. Forel for Environmental and Aquatic Sciences and Institute for Environmental Sciences, University of Geneva, 66 boulevard Carl-Vogt, Geneva 1211, Geneva, Switzerland

**Keywords:** chytrid infection, heterocyst, host growth, nitrogen availability, nitrogen fixation

## Abstract

Chytrids infect and kill various phytoplankton. Studies of chytrids infecting cyanobacteria and microalgae have focused only on a few host–parasite systems (hosts mainly *Planktothrix* and *Asterionella*). Here we focus on a newly isolated and recently described chytrid that infects the nitrogen-fixing filamentous cyanobacterium *Dolichospermum*. This species specializes in infecting heterocyst cells only and may therefore affect the nitrogen fixation process. We performed infection experiments where *Dolichospermum* was exposed to the chytrid under nitrogen-limited and nitrogen-replete conditions and quantified the effects of infection on host nitrogen fixation and growth. Chytrid infection strongly decreased host growth under nitrogen-limited conditions, but not under nitrogen-rich. This was because the cyanobacterium could only obtain nitrogen from its partially infected heterocysts, which, despite the parasitism, retained some capacity for nitrogen fixation under nitrogen-limited conditions, but at a reduced level. Host filaments partially compensated for chytrid infection by increasing nitrogen fixation rates nearly 8-fold in the remaining heterocysts that survived infection. Nitrogen fixation rates were reduced by ⁓50% in infected *Dolichospermum* when normalized to biovolume, compared to uninfected controls. This reduction in the supply of nitrogen through nitrogen fixation and cyanobacterial development suggests that chytrid parasites may shape cyanobacterial bloom development and nitrogen fixation in nature.

## INTRODUCTION

Chytridiomycota is a phylum of a group of fungal parasites that infect a variety of organisms. While chytridiomycosis caused by *Batrachochytrium* spp. has caused global amphibian declines (Berger *et al.,* 1998; Fisher and Garner, 2020; Kilpatrick *et al.,* 2010; Lips, 2016; Longcore *et al.,* 1999; Martel *et al.,* 2013; O’hanlon *et al.,* 2018), parasitic chytrids exhibit broader ecological significance, e.g. through infections of phytoplankton. Chytrids parasitising phytoplankton has been described for decades and is now known to be a common biotic interaction in the aquatic environment (Ibelings *et al.,* 2004). They are characterized by the production of motile zoospores, which are recognizable by the possession of a single flagellum to propel swimming motion. Being diverse and ubiquitous, and accounting for a large part of aquatic parasitism, chytrid infection has recently gained increased attention because of its broad role in aquatic ecosystem dynamics (Frenken *et al.,* 2017b).

Cyanobacteria are important primary producers (Sánchez-Baracaldo *et al.,* 2022), accounting for ~10% of global primary production (Rousseaux and Gregg, 2013). Some cyanobacteria are also able to fix atmospheric di-nitrogen, a trait absent in eukaryotic phytoplankton (Latysheva *et al.,* 2012). Roughly 50% of global nitrogen fixation is performed by cyanobacteria (Latysheva *et al.,* 2012). Cyanobacteria use different strategies for this, such as the development of special differentiated cells, called heterocysts (Muro-Pastor and Hess, 2012). Heterocyst production is promoted when nitrogen sources like ammonium or nitrate in the aquatic environment—hereafter referred to as fixed nitrogen sources—are low. The ability to form heterocysts has tentatively been dated to the very beginning of cyanobacterial evolution (Schopf, 2002; Berman-Frank *et al.,* 2003).

In 1963, Canter discovered and described a chytrid capable of infecting the heterocysts of *Aphanizomenon flos-aquae* (Canter, 1963). Much later, others observed chytrid infection of the heterocyst of *Anabaena smithii* (Takano *et al.,* 2008). To our knowledge, these are the only two previous studies that have described chytrid associations with cyanobacterial heterocysts. Recently we isolated another chytrid capable of infecting heterocysts of the cyanobacterial genus *Dolichospermum*, named Dol-Heterocyst-01. Phylogenetic analysis (Xu *et al.,* 2025) has placed its origin in a novel clade that is a sister clade to the *Lobulomycetales*, a new order within the Chytridiomycota (Simmons *et al.,* 2009).

The effects of chytrid infection on nitrogen fixation and growth of the cyanobacterial host so far has not been investigated. Infections of cyanobacteria by parasitic chytrids generally lead to death of the infected cells (Ibelings *et al.,* 2004). Chytrid infection of heterocysts, however, might have a different outcome for the host filament. This may depend on the extent to which infection affects the nitrogen fixation process, and the exchange of nitrogen fixed by the heterocyst with the vegetative cells in the filament, modulated by the availability of fixed nitrogen in the environment, which routinely will be taken up by the vegetative cells. In the presence of sufficient nitrogen, even a complete shutdown of nitrogen fixation through chytrid infection should have a limited, if any, impact on host growth and survival.

This study consisted of two parts, designed to assess the impact of the recently discovered heterocyst infecting chytrid. In Part 1 we tracked host growth, heterocyst dynamics and infection progression over 18 days in N-limited and N-rich media. In Part 2 we quantified nitrogen fixation rates via the acetylene reduction assay (ARA), performed at the end of the experiment providing a final impression of the impairment of nitrogen fixation caused by chytrid parasitism. Both parts used the same replicate cultures to link temporal dynamics with endpoint measurements, ensuring consistency between the parts and reducing inter-experimental variability. We find that heterocyst infection reduced *Dolichospermum* growth rate, but only in the absence of fixed nitrogen and that the infected filaments partially compensated for the loss of heterocysts by greatly increasing the nitrogen fixation rate per heterocyst.

## MATERIALS AND METHODS

### Cyanobacteria and chytrid cultures

We isolated a heterocystous cyanobacteria species belonging to the genus *Dolichospermum* from Lake Stechlin (Germany) in 2018. It was maintained in batch culture in modified Z8 medium (without a nitrogen source, hereafter refer to as N-limited medium), in contrast to standard Z8 medium (which contains ammonium, hereafter refer to as N-rich). For media composition details see Supplementary Material: Table . Incubation was carried out in a Multitron incubator (INFORS HT, Switzerland). Cultures were grown at a constant temperature of 22°C, under a 16:8 h light: dark cycle and a mean irradiance level of 59 μmol photons m^−2^ s^−1^, with shaking at 100 rpm. The chytrid strain, Dol-Heterocyst-01, newly discovered and isolated from Lake Stechlin in 2018, was used to infect cultures of *Dolichospermum* in the experiments. This chytrid parasite is only capable of infecting the heterocyst cells of its *Dolichospermum* host (Xu *et al.,* 2025). The parasite was maintained in culture by the weekly addition of host filaments.

### Experimental setup and design

A 2 × 2 factorial experiment was performed to investigate the effects of chytrid infection and nitrogen supply on cyanobacterial host growth. Two media types (N-rich and N-limited media) were crossed with two infection levels (infected and uninfected). The cyanobacteria host culture was acclimated to N-limited medium and was used in the experiment for both N-treatments, i.e. without prior acclimation to the N-rich medium. This way we ensured that at the start of the experiment all *Dolichospermum* had the same opportunity to fix N_2_, so that the differences in heterocyst density, linked to this infection levels, and finally rates of N_2_ fixation were caused by the experimental differences experienced during the 18-day incubation. Fifteen mL of uninfected dense host culture was added to 105 mL corresponding medium in a 250 mL Erlenmeyer flask (Greiner Bio One, Item No.: 658195), to obtain a desired host biovolume of 17 nL mL^−1^. The desired host biovolume of 17 nL mL^−1^ (= mm^3^ L^−1^) was chosen based upon our lab’s experience that this would ensure sufficient host density to sustain chytrid infection, while avoiding collapse of the batch host culture toward late stages. Moreover, although a value of 17 nL mL^−1^ would be higher than the biovolume observed in lakes, it is not far outside the upper range found in the literature for *Anabaena/Dolichospermum* blooms, e.g. 14 mm^3^ L^−1^ in an Australian pond (Mitrovic *et al.,* 2011), or 10 mm^3^ L^−1^ for a Dutch lake (Piel *et al.,* 2024), and >10 mm^3^ L^−1^ in a Japanese reservoir (Tsujimura and Okubo, 2003).

For each of the four treatments, we created 8 replicates containing 120 mL of culture per flask, resulting in a total of 32 experimental units. For the “infected” treatments, a filtered chytrid zoospore suspension, made by passing 2 mL of infected *Dolichospermum* culture through a sterile serological pipet filled with glass fiber, was added to the flasks. The experiment lasted for 18 days. At each sampling time, flasks were randomly re-assigned positions to avoid biases caused by gradients in light or temperature within the incubator. Flask position within the incubator was set using a random number generator (https://www.random.org/lists/).

### Sample preparation and analysis for host growth and chytrid infection

Host growth and chytrid infection measurements were analyzed using a newly developed imaging method. Two mL of culture from each flask was sampled every second day, fixed with glutaraldehyde (Sigma-Aldrich, Switzerland) with a final concentration of 0.5%, and stored at 4°C for later analysis. Prior to imaging, the gas vesicles of the host cells needed to be collapsed to enable sedimentation. For this a 2 mL syringe filled with the cyanobacterium was hit 5 times against the bench; the resulting shock wave collapsed the gas vesicles and made the filaments settle. Identification and counting of all chytrid sporangia that developed during the incubation allows us to claim that the shock wave did not dislodge sporangia from the heterocysts since loose sporangia were not observed.

Imaging analysis was composed of two steps including a plate reading process with Cytation-3 (BioTek, Winooski, VT, USA) and subsequent picture analyzing process with open-source software ImageJ (Schindelin *et al.,* 2012). In brief, 96 black bottom well-plates (Greiner Bio one Item No: 655096) containing 300 μL samples in duplicate were set-up 24 h prior to reading, to allow filament sedimentation. After, plates were mounted and imaged at 4x magnification. For host growth measurement, images were taken under the chlorophyll and phycoerythrin autofluorescence channel (586 nm excitation and 647 nm emission), which yields high quality images with bright filaments against a dark background. For infection dynamics, images were taken at 20× magnification in the bright field channel (black and white). Chytrid attached zoospores and sporangia were clearly visible and could be counted from the images. Subsequent image analysis with ImageJ software was done to acquire total length of all the filaments from each well using the plugin AnalyzeSkeleton 3.1.3 (Arganda-Carreras *et al.,* 2010). For more details about the image analysis process, see Wierenga *et al.* (2022).

Host growth was represented by following the development of biovolume of all filaments in a sample, calculated using ImageJ based on the total filament length and a single diameter estimate. Single cell diameter was calculated by averaging the width of 50 randomly selected single cells for all the treatments from days 0, 6, 12 and 18. Biovolume was calculated based on the cylinder volume equation: V = π(d/2)^2^h, where d is the cell diameter and h is the total filament length. Average filament length was calculated via dividing the total filament length by number of filaments counted. Growth rates for each condition were calculated as the slope of the graph in which logistically transferred growth of biovolume was fitted against corresponding time. Growth rates of *Dolichospermum* from each condition were calculated by fitting the data with the “SLOPE” function from excel (Microsoft, USA), which is defined with the equation below:


\begin{align*}b=\frac{\sum \left(x-\overline{x}\right)\left(y-\overline{y}\right)}{\sum{(x-\overline{x} )}^2} \end{align*}

where b is the slope, x is the time point (day number) and y is the log-transformed biovolume. The “barred terms” in regression formulas denote sample means of the corresponding variables. Growth rates were calculated on basis of biovolume over the full course of the experiment. Note that these are not standard exponential growth rates because biovolume may increase and then decrease as a response to infection over the course of the experiment.

Chytrid infection data were estimated by checking 100 random filaments from each flask. Both prevalence and intensity of infection were calculated. Prevalence of infection was calculated as the number of infected heterocysts divided by the total number of heterocyst counted, and the intensity of infection as number of infections found divided by the total number of infected heterocysts. These numbers differ because one heterocyst can have more than one infection. Infections were scored both as attached zoospores and as developing or mature sporangia. Empty sporangia were excluded from the count. Heterocyst number was again obtained by randomly checking 100 filaments from each flask for the presence of heterocysts.

### Nitrogenase activity

To evaluate the effect of chytrid infection and nitrogen availability on nitrogenase activity, an ARA was performed at the end of the experiment following protocols by Hardy *et al.* (1968). Compared to the ^15^N_2_ stable isotope method, which is recognized to be the most reliable method to estimate nitrogenase activity (as it directly measures N fixation), the ARA assay is faster and more cost-efficient. The principle of the ARA assay is that if acetylene (C_2_H_2_) is present, the nitrogenase enzyme reduces acetylene (C_2_H_2_) to ethylene (C_2_H_4_). Therefore, the production of C_2_H_4_ reflects nitrogenase activity, based on the theoretical conversion ratio of the reaction’s biochemistry.

Briefly, 5 mL of each culture was incubated with 10% acetylene in a 20 mL vial for 3 h, under the same experimental conditions. After incubation, the excess acetylene was removed by adding 2 mL of 1 M ammoniacal cuprous chloride solution into the exetainers, reacting with acetylene and forming a red precipitate. To complete the reaction, the exetainers were shaken for 24 h on an orbital bench-top shaker. Afterwards, 10 mL of gas samples were collected into 4.5 mL exetainers and sent to Cambridge Refrigeration Technology Limited (Cambridge, UK) for analysis. Ethylene production was assessed by measuring the height of the ethylene peak with a Photovac 10S50 gas chromatograph (PerkinElmer, Inc. Waltham, US) equipped with a Carbopac Butylated Hydroxy Toluene (BHT) column and pre column (Sigma-Aldrich Co., Burlington, US). Pure ethylene standards were made based on the same procedure described above. Results of the nitrogenase activity measured as μmol C_2_H_4_ L^−1^ h^−1^ were converted to N input (mmol or μmol N L^−1^ h^−1^) using the theoretical conversion ratio 3 (Rice and Paul, 1971), and were calculated at biovolume and heterocyst levels, respectively.

### Statistical analyses

We fit a two-way ANOVA to explain variation in host growth and nitrogen fixation rates using chytrid infection, nitrogen availability and their interaction as predictors. Significant differences between treatments were identified by Tukey Honestly Significant Difference (HSD) *post hoc* tests. All data analysis and subsequent graphical representation were performed using GraphPad Prism 9.3.1 (471).

## RESULTS

### Chytrids reduce host growth in the absence of nitrogen

**Fig. 1:**
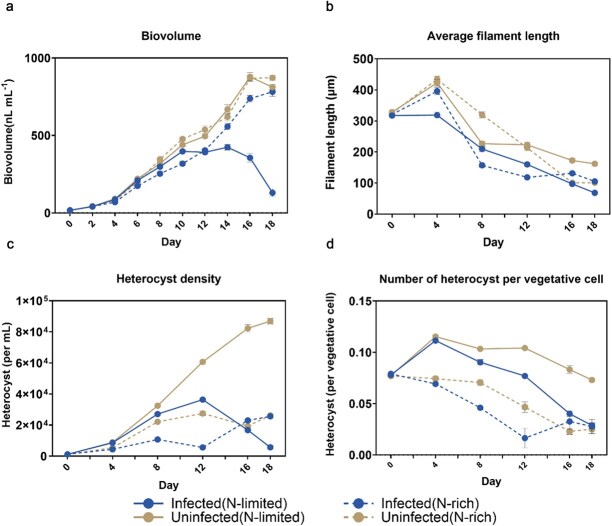
Host density dynamics and heterocyst number changes over time for infected and uninfected treatments in N-limited (solid lines) and N-rich (dotted lines) media. (a) Biovolume dynamics, (b) Average filament length changes, (c) heterocyst density (mL^−1^) changes over time. (d) Heterocysts per vegetative cell. Error bars indicate standard errors.

**Fig. 2:**
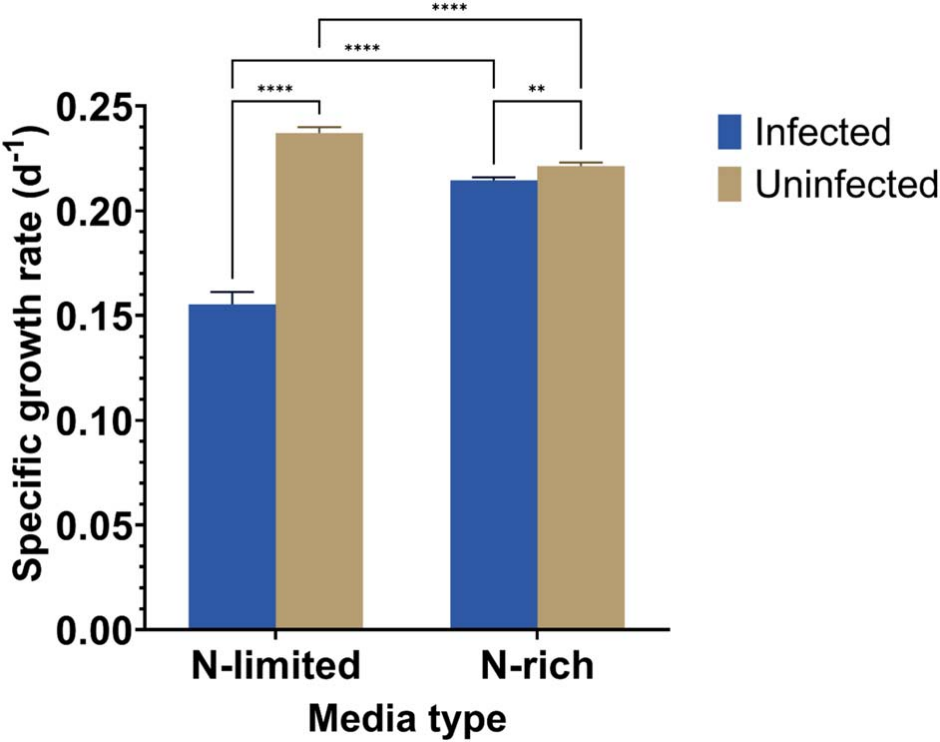
Growth rates calculated on basis of changes in *Dolichospermum* biovolume over time for infected and uninfected cultures in both N-limited and N-rich media. Note that these are not exponential growth rates because growth was not exponential over the entire time period, especially as a result of infection in N-free cultures (see Fig. 1(a)). Error bars indicate standard errors, number of stars represent significance levels. ^****^P < 0.0001, ^*^P < 0.05, ns: not significant, *P* > 0.05.

Chytrid infection reduced the biovolume of the cyanobacterial host over 18 days, but only in N-limited medium. In this treatment biovolume dropped steeply after day 10. In N-rich medium the development of biovolume over time was similar between infected and uninfected cultures (Fig. 1(a); Supplementary Material: Table ). In N-limited medium, however, the biovolume development in infected cultures lagged behind uninfected from 10 day onwards and dropped steeply after day 14. Host growth rates therefore were much lower in the infected N-limited than N-rich treatment, but other treatments also differed to a certain degree (Fig. 2). Host filament length decreased throughout the experiment in both infected and uninfected treatments. In N-limited medium, filaments in infected treatments were significantly shorter than uninfected controls by day 18 (*P* < 0.0001; Fig. 1(b)). In N-rich medium, no significant difference in filament length was detected between infected and uninfected treatments (*P* > 0.05; Supplementary Material: Table ).

### N-rich conditions and infection reduce presence of heterocysts

Heterocysts were found in all treatments, including those with ample fixed N in the growth medium, where approximately one in 13 cells were identified as a heterocyst. Heterocyst formation was heavily affected by chytrid infection (Fig. 1(c) and (d)). In uninfected N-limited media, heterocyst density closely tracked overall host density (Fig. 1(a) and (c)). Conversely, in N-rich media, heterocyst density decreased over time due to reduced selective pressure to maintain nitrogen fixation (Fig. 1(c)). The number of heterocysts per vegetative cell first increased for both infected and uninfected treatments in the N-limited medium but decreased afterwards, and the decline was faster in infected than in uninfected treatments (Fig. 1(d)). In N-rich medium, the heterocyst frequency decreased in both infected and uninfected cultures from the beginning. The infected N-limited and N-rich cultures converged on very similar heterocyst frequencies at the end, while the uninfected N-limited cultures showed a frequency approximately twice as high (Fig. 1(d)).

### Prevalence and intensity of infection increase to higher levels in N-limited treatments

**Fig. 3:**
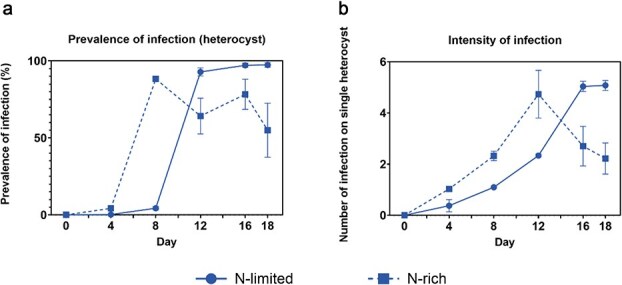
Prevalence and intensity of infection in the infected treatments. (a) The prevalence of infection (%) of heterocysts, (b) intensity of infection i.e. the mean number of chytrids infecting a single heterocyst. Solid and dotted lines representing N-limited and N-rich conditions, respectively. Error bars indicate standard errors.

The prevalence of infection increased from zero to nearly 100% in N-limited medium by the end of the experiment, with a steep increase between days 8 and 12. It increased earlier in N-rich medium, between day 4 and 8, but decreased from a peak of ⁓90% to ⁓60% toward the end (Fig. 3(a)). The mean number of chytrids infecting each heterocyst (the intensity of infection) increased steadily to approximately five in the N-limited medium. As with infection prevalence, it increased earlier in N-rich medium but decreased to approximately two infections per heterocyst toward the end (Fig. 3(b)). Therefore, at the end of the experiment, both the prevalence and the intensity of infection were higher in the N-limited treatments.

### Chytrids reduce N_2_-fixation in N-limited cultures but increase fixation rates per heterocyst

**Fig. 4:**
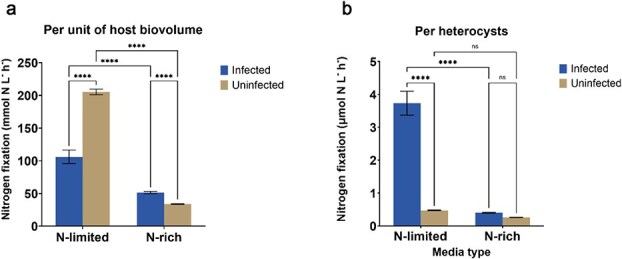
Nitrogen fixation rates (a) per unit of host biovolume (1 mL) and (b) per heterocyst differed strongly based on nitrogen availability and infection status. Individual heterocysts in infected N-limited cultures fixed nearly seven times as much nitrogen as those in uninfected N-limited cultures, even though uninfected cultures fixed more nitrogen overall. Error bars indicate standard errors, and the number of stars represent significance levels. ^****^P < 0.0001, ns: not significant, *P* > 0.05.

N_2_-fixation rates, per unit of host biovolume, in N-limited conditions unsurprisingly surpassed those under N-rich conditions, although N_2_-fixation was not reduced to zero in the presence of fixed nitrogen. Overall, chytrid infection halved nitrogen fixation rates per unit host biovolume (1 mL) in the N-limited medium (Fig. 4(a); Supplementary Material: Table SIII). Interestingly nitrogen fixation rates per heterocyst increased nearly 8-fold in the infected N-limited cultures in comparison to the uninfected ones (Fig. 4(b)). In N-rich medium, N_2_-fixation was similar or slightly higher in the infected treatment, while the N-fixation rate per heterocyst was not significantly different (Fig. 4(a); Supplementary Material: Table SIV).

## DISCUSSION

Both cyanobacterial N_2_-fixation (Fay, 1992; Bothe *et al.,* 2010; Latysheva *et al.,* 2012) and to a lesser extent cyanobacteria infecting chytrids (Ibelings *et al.,* 2004; Gerphagnon *et al.,* 2015; Gleason *et al.,* 2015; Frenken *et al.,* 2017a) are topics that are widely represented in the literature, but specialized chytrids infecting heterocysts remain poorly understood. In this paper we tested the consequences of heterocyst infections for the process of N_2_-fixation, and the growth of diazotrophic cyanobacterial hosts. Our experimental results demonstrate that negative effects on host growth are restricted to conditions where fixed sources of nitrogen are absent in the growth medium. Not unexpected, but these clear outcomes of infection by a specialized chytrid from a controlled experiment is novel and of interest.

Parasitic chytrids typically harm hosts, often lethally, and high infection prevalence can decimate host populations (Ibelings *et al.,* 2004; Rosenblum *et al.,* 2010; Ibelings *et al.,* 2011; Gerphagnon *et al.,* 2013). Heterocysts increase in frequency under nitrogen limitation (Zulkefli and Hwang, 2020), to provide the cyanobacterial filaments with sufficient nitrogen, leading us to hypothesize that chytrid impacts would be restricted to nitrogen-deprived conditions. As long as a fixed nitrogen source remains present in the environment heterocysts are less abundant (relevant), and chytrid infection of these heterocysts should have little effect on host growth, since vegetative cells continue to take up nitrogen from their surroundings (Fig. 1(a) and (c)). A reduced presence of heterocysts also restricts the possibility for infection to spread. We find strong evidence for these expectations in our study. We show that the growth of host cyanobacteria was strongly reduced by heterocyst-infecting chytrids, but only when the host was experiencing nitrogen depletion in its environment and therefore had to rely on N_2_-fixation for its nitrogen supply.

Host cell density drives chytrid infection dynamics, as parasites require hosts to complete their life cycle (May *et al.,* 1981; Ibelings *et al.,* 2004). Transmission is density-dependent: high host density increases infection prevalence by aiding zoospore-host encounters. Environmental factors such as light intensity, temperature and nutrient availability modulate host density as well as various parts of the chytrid life cycle, and thus may alter chytrid fitness directly or indirectly. For our specialized chytrid, nitrogen availability directly modulated heterocyst density (Fig. 1(c)), the only target cells for infection. Under nitrogen depletion, increased heterocyst density elevated encounter rates, thus boosting infection prevalence and intensity (Fig. 3(a) and (b)).

Infections occur when a viable zoospore encounters, attaches and penetrates a susceptible host cell. Figure 1(d) shows that from equal start at inoculation the heterocyst frequency declines faster under N-rich than N-limited conditions. In both N-rich and N-limited conditions heterocysts will decrease under impact of infection, but the rate at which new heterocysts are produced is lower under N-rich conditions, in particular when the energetically most attractive form of N, ammonium, is freely available (Sanz-Alférez and Del Campo, 1994). Heterocystous cyanobacteria have been shown to be able to perform some nitrogen fixation in the presence or of a source of fixed nitrogen (Inomura *et al.,* 2018; Inomura *et al.,* 2019).

Hence, when being transferred at the start of the experiment to an N-rich environment, the number of heterocysts is expected to decline, even in the absence of infection. This has been known for decades, e.g. Murry and Benemann (1979) demonstrated experimentally that for *Anabaena cylindrica* repeated transfers into ammonium rich medium drove heterocysts from ~ 5% to < 1% of total cell number over five serial transfers. So, this well studied physiological response explains an overall reduction in heterocyst frequency in N-rich compared to N-limited cultures. On top of this, the heterocyst frequency declined faster in infected than in uninfected cultures. The combination of both scaling down heterocyst production and removing heterocysts through infection contributed to the observed fast decline under N-rich, infected conditions. Under N-limited conditions differentiation of heterocysts is maintained at the initial level and only infection explains the steeper drop—in this case compared to N-limited/uninfected.

Zoospores typically rely on chemotaxis to find a host cell. In the case of our specific host—parasite system, the zoospores need to find their target cells—strictly heterocysts—in an environment dominated by vegetative *Dolichospermum* cells. The lower the heterocyst frequency the larger the likelihood that a zoospore cannot locate a target cell within its infective lifetime. In an earlier study we report an infective lifetime of zoospores of a *Planktothrix rubescens* infecting chytrid to be 2.71 days at 17°C (Wierenga *et al.,* 2022). Zoospores, unless the host density is very high, do not rely on random encounters with their host cell, but actively swim toward them using chemotaxis, i.e. detecting gradients in exudates given off by the host (Van Den Wyngaert *et al.,* 2013). In the Wierenga *et al.* study cited above the maturation time of sporangia was found to be between 2 and 3 days, and decreased with increasing temperature.

Earlier, Holfeld based upon intensive field studies proposed that living non-host algae interfere with the process of host detection by swimming zoospore, i.e. non-host algae constitute a signal-dilution leading to a reduction in successful encounters (Holfeld, 2000). Infection scales with the proportion of suitable host cells in the community. This mechanism protects hosts when they are embedded in diverse phytoplankton assemblage. When we extend this principle to vegetative cells that shield the signal from the target heterocysts this could perhaps explain why the prevalence and intensity of infection decreases faster under N-rich than N-limited conditions (Fig. 3(a)). After transfer to N-rich medium heterocysts gradually go down over a few generations of *Dolichospermum* growth. It just becomes increasingly unlikely that zoospores can find and infect these ever-rarer heterocysts under N-rich conditions (Fig. 1(c) and (d)). In other words, the proportion of target host cells—heterocysts—of the total number of cells decreases over time. Moreover, Fig. 2 shows that the growth rate of infected N-rich cultures exceeds the growth rate in infected N-limited ones. Thus, during the 18 days more vegetative cells are produced in N-rich medium, and logically this strengthens the “shielding” of heterocysts among the vegetative cells.

**Fig. 5:**
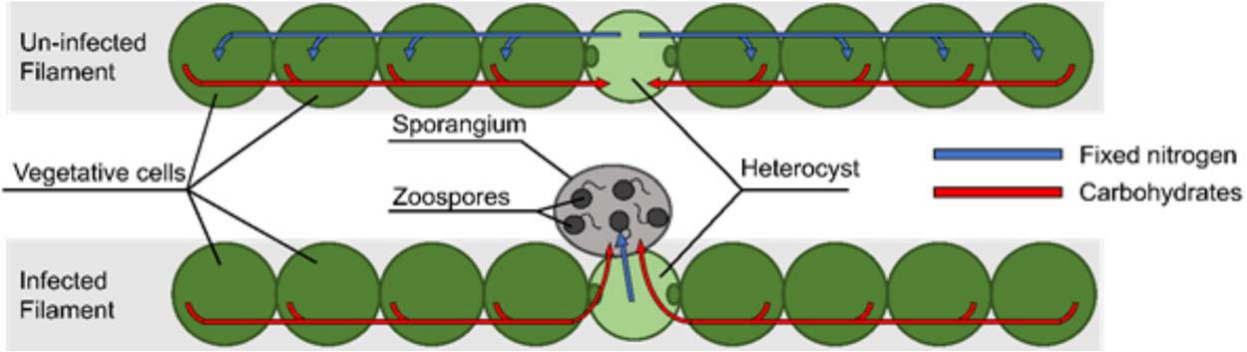
Schematic diagram model of infected and uninfected *Dolichospermum* filaments showing the possible mechanism of internal changes of *Dolichospermum* cells under chytrid infection, both vegetative cells and heterocyst.

Our nitrogen fixation experiments showed that under nitrogen-limited conditions, chytrid infection reduced nitrogen fixation rate per unit host biovolume. However, remaining uninfected heterocysts exhibited an ~ 8-fold increase in individual nitrogenase activity, suggesting some form of a regulatory response to compensate for lost N₂-fixation. A possible sequence of events could work as follows. The development of the chytrid zoospore into a mature sporangium depends on the host’s supply of nutrients to which the infecting chytrid gains access. For hosts under nitrogen-limited conditions, the host filament relies on the heterocysts to supply nitrogen. Heterocysts receive nutrients and energy from neighboring vegetative cells, to achieve nitrogen fixation, a highly resource-expensive process. When an increasing number of heterocysts get infected, in order to maintain a sufficient supply of nitrogen, *Dolichospermum* filaments would have to allocate more resources to the remaining few heterocysts (Fig. 5). This could then result in an increased nitrogen fixation rate per heterocyst (Fig. 4(b)), and as such to continue to provide nitrogen for the filament. Host filament growth can continue for a while, also using already existing nitrogen supplies in the vegetative cells, but under severe infection N-limited cultures cannot escape termination. Hence the sharp drop in *Dolichospermum* biovolume in the infected, N-limited treatment in the final days of the incubation (Fig. 1(a)).

From our observations it is clear that multiple sporangia can develop on a single heterocyst. Something that seems unlikely if the resources would only come from that single heterocyst. However if we take into account that heterocysts receive carbohydrates from adjacent vegetative cells, and can fix enough nitrogen for multiple vegetative cells, it seems much more probable that multiple sporangia can develop since the developing fungi do not just consume what is available in the infected heterocyst, but continue to consume what is being transported to the heterocyst by the vegetative cells, and whatever nitrogen is still being fixed by the heterocyst (Fig. 5).

This aligns with systemic resource redistribution in cyanobacteria, such as plasticity in heterocyst spacing under nitrogen stress (Latysheva *et al.,* 2012). or energy redirection under biotic stress (Kuznecova *et al.,* 2020). Furthermore, the concept of “metabolic compensation” in uninfected tissues is well-supported in other host–parasite systems. For instance, parasitized legumes upregulate nitrogen fixation in uninfected nodules to compensate for pathogen-driven losses (Zahran, 1999), and coral hosts redirect energy to unaffected polyps following tissue damage (Palmer, 2018). Analogously, our finding that uninfected heterocysts maintain nitrogenase activity in N-rich media (Fig. 4(b)) suggests a host strategy to buffer against parasitic losses, even at the cost of reduced growth. While speculative, these parallels highlight the potential for cross-system generalities in parasite-mediated resource dynamics. Future studies integrating transcriptomics or stable isotopes could directly test whether cyanobacteria employ analogous metabolic reprioritization mechanisms.

This interaction between chytrid and heterocyst might also introduce a novel pathway for nitrogen flow in trophic food web, akin to the mycoloop, a trophic link via chytrid zoospores from large inedible algae to *Daphnia* grazers (Kagami *et al.,* 2007). N_2_ fixing cyanobacteria such as *Dolichospermum* have been often reported to produce blooms in less productive lakes (Sterner *et al.,* 2020; Reinl *et al.,* 2021). Nitrogen fixed by filamentous cyanobacteria cannot be efficiently used at higher trophic levels, since they are sometimes considered to be trophic dead ends (Porter, 1980). With chytrid infection of heterocysts, nitrogen fixed could be introduced to the lake’s nitrogen cycle, through consumption of chytrid zoospores by zooplankton (Agha *et al.,* 2016). Our results clearly show that chytrid infection of heterocysts interferes with the process of N_2_-fixation. It is therefore tempting to speculate what this may mean for N_2_-fixation at the lake scale. However, for this we would need data on the duration and prevalence of infection in lakes. Since these data are missing, reliable extrapolation from the results of our study to whole systems is not yet possible.

To clarify the mechanistic basis of host–parasite interactions, future work could (i) quantify zoospore maturation and release timing using live-cell imaging, (ii) investigate the genetic and metabolic basis of the host’s compensatory nitrogenase response via transcriptomics, (iii) measure resource transport between vegetative cells and infected heterocysts using stable isotopes to track carbon and nitrogen fluxes, (iv) assess infection prevalence and duration in natural lakes to determine ecological relevance.

## CONCLUSION

This study shows that chytrid parasites that are specialized in infecting heterocysts of N_2_-fixing cyanobacteria affect host nitrogen fixation rates and growth. Unlike other chytrid parasites, heterocyst-associated chytrids are a threat for their host primarily under nitrogen depleted conditions. However, hosts appear to possess a regulatory mechanism to partly compensate for heterocyst losses by substantially increasing the activity of the remaining uninfected heterocysts. Interactions between heterocysts and chytrids may introduce a novel pathway in nitrogen cycling in aquatic ecosystems. Much remains to be studied, however on this novel and specialized chytrid and its N_2_-fixing cyanobacterial host.

## Supplementary Material

Supplementary_material_fbaf054
