# Supplementary material for: Heterocyst-infecting chytrid parasites reduce nitrogen fixation and host growth under nitrogen-limiting conditions in the cyanobacterium Dolichospermum sp
Source: J Plankton Res. 2025 Oct 8;47(6):fbaf054. doi: 10.1093/plankt/fbaf054 (PMC12507009; doi:10.1093/plankt/fbaf054)
Supplement: Supplementary_material_fbaf054 [file supplementary_material_fbaf054.docx]

**SUPPLEMENTARY MATERIAL**

Table SⅠ. Media composition of Z8 and Z8X

| Z8(X) medium composition | | | |
| --- | --- | --- | --- |
| Macronutrients (for 1L) | | Micronutrients (for 1L) | |
| CaCl_2_ (for Z8X) | 0.037 g | Na_2_WO_4_·2H_2_O | 0.0033 mg |
| MgSO_4_ | 0.025 g | (NH_4_)_6_Mo_7_O_24_·4H_2_O | 0.0088 mg |
| K_2_HPO_4_ | 0.031 g | KBr | 0.0121 mg |
| Na_2_CO_3_ | 0.021 g | KI | 0.0083 mg |
| FeCl_3_ | 0.0028 g | ZnSO_4_·7H_2_O | 0.0287 mg |
| Na_2_EDTA | 0.0039 g | Cd(NO_3_)_2_·4H_2_O | 0.0155 mg |
| NaNO_3_ (for Z8) | 0.467 g | Co(NO_3_)_2_·6H_2_O | 0.0146 mg |
| Ca(NO_3_)_2_ (for Z8) | 0.059 g | CuSO_4_·5H_2_O | 0.0125 mg |
|  |  | (NH_4_)_2_Ni(SO_4_)_2_·6H_2_O | 0.0198 mg |
|  |  | Cr(NO_3_)_3_·7H_2_O | 0.0041 mg |
|  |  | Al_2_(SO_4_)_3_K_2_SO_4_·2H_2_O | 0.0474 mg |
|  |  | V_2_O_3_ | 0.00089 mg |
|  |  | H_3_BO_3_ | 0.31 mg |
|  |  | MnSO_4_·4H_2_O | 0.233 mg |

Table SⅡ. Two-way ANOVA results for biovolume growth rates (day 0-18)

| Factor | df | F ratio | p-value | % of total variation |
| --- | --- | --- | --- | --- |
| Infection | 1 | 1368 | p<0.0001 | 50.54 |
| Nitrogen | 1 | 328.6 | p<0.0001 | 12.13 |
| Infection x Nitrogen | 1 | 982.7 | p<0.0001 | 36.30 |
| Residual | 28 |  |  | 1.03 |

Table SⅢ. Two-way ANOVA results for nitrogen fixation at biovolume level

| Factor | df | F ratio | p-value | % of total variation | |
| --- | --- | --- | --- | --- | --- |
| Infection | 1 | 419.0 | p<0.0001 | | 9.33 |
| Nitrogen | 1 | 3193 | p<0.0001 | | 71.09 |
| Infection x Nitrogen | 1 | 851.2 | p<0.0001 | | 18.95 |
| Residual | 28 |  |  | | 0.63 |

Table SⅣ. Two-way ANOVA results for nitrogen fixation at heterocyst level

| Factor | df | F ratio | p-value | % of total variation | |
| --- | --- | --- | --- | --- | --- |
| Infection | 1 | 698.9 | p<0.0001 | | 33.70 |
| Nitrogen | 1 | 759.5 | p<0.0001 | | 36.62 |
| Infection x Nitrogen | 1 | 587.3 | p<0.0001 | | 28.32 |
| Residual | 28 |  |  | 1.36 | |

Table SⅤ. Linear mixed model results for filament length change dynamic

|  | length |  |  |  |
| --- | --- | --- | --- | --- |
| Predictors | Estimates | CI | p | df |
| (Intercept) | 68.31 | 53.14 – 83.48 | <0.001 | 9.21 |
| nitrogen [N-rich] | 36.91 | 15.45 – 58.37 | 0.004 | 9.21 |
| infected [UI] | 93.55 | 81.94 – 105.15 | <0.001 | 1590 |
| nitrogen [N-rich] × | -97.91 | -114.32 – -81.50 | <0.001 | 1590 |
| infected [UI] |  |  |  |  |
| Random Effects | |  |  |  |
| σ2 | 7000.02 |  |  |  |
| τ00 replicate | 111.25 |  |  |  |
| ICC | 0.02 |  |  |  |
| N replicate | 8 |  |  |  |
| Observations | 1600 |  |  |  |
| Marginal R2 / Conditional R2 | 0.137 / 0.151 | |  |  |

We counted the lengths of 100 filaments from half the replicates belonging to each treatment. We fit a linear mixed model to explain variation in filament length on the final day of the experiment using chytrid infection, nitrogen availability and their interaction as fixed-effect predictors and replicate as a random intercept. p-values were calculated using the Kenward-Roger degrees of freedom approximation for mixed models. Very similar coefficient estimates and uncertainties were obtained when we used a Bayesian hierarchical model with weakly informative priors instead (not shown here).
